# Supplementary material for: FDG-PET/CT in the Monitoring of Lymphoma Immunotherapy Response: Current Status and Future Prospects
Source: Cancers (Basel). 2023 Feb 7;15(4):1063. doi: 10.3390/cancers15041063 (PMC9954669; doi:10.3390/cancers15041063)
Supplement: Supplementary file 1 [file cancers-15-01063-s001.zip › Updated Supplementary Material.pptx]

## Slide 1
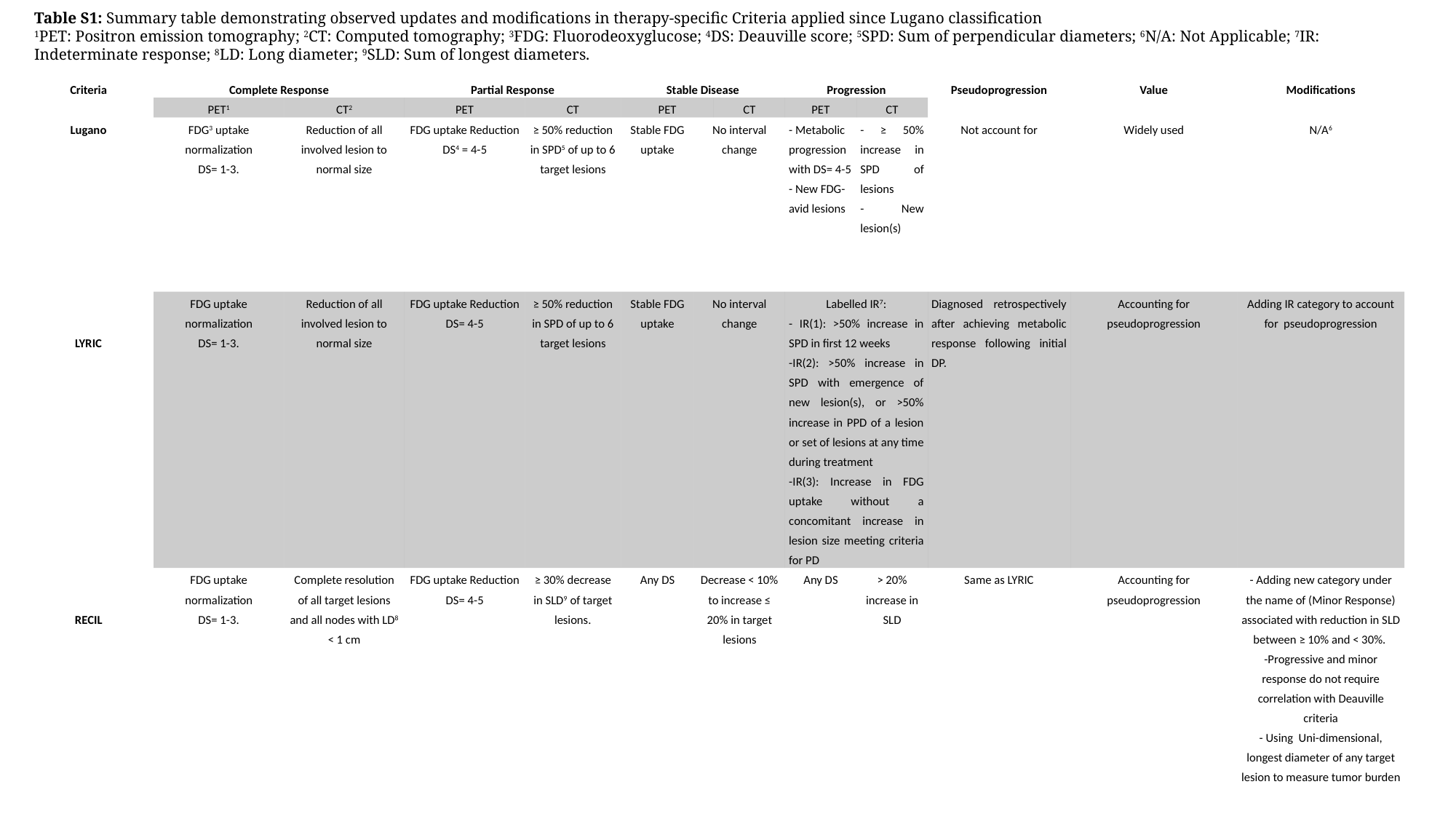

Table S1: Summary table demonstrating observed updates and modifications in therapy-specific Criteria applied since Lugano classification
1PET: Positron emission tomography; 2CT: Computed tomography; 3FDG: Fluorodeoxyglucose; 4DS: Deauville score; 5SPD: Sum of perpendicular diameters; 6N/A: Not Applicable; 7IR: Indeterminate response; 8LD: Long diameter; 9SLD: Sum of longest diameters.
| Criteria | Complete Response | | Partial Response | | Stable Disease | | | Progression | | Pseudoprogression | Value | Modifications |
| --- | --- | --- | --- | --- | --- | --- | --- | --- | --- | --- | --- | --- |
| | PET1 | CT2 | PET | CT | PET | | CT | PET | CT | | | |
| Lugano | FDG3 uptake normalization DS= 1-3. | ­Reduction of all involved lesion to normal size | FDG uptake Reduction DS4 = 4-5 | ≥ 50% reduction in SPD5 of up to 6 target lesions | Stable FDG uptake | No interval change | | - Metabolic progression with DS= 4-5 - New FDG-avid lesions | - ≥ 50% increase in SPD of lesions - New lesion(s) | Not account for | Widely used | N/A6 |
| LYRIC | FDG uptake normalization DS= 1-3. | ­Reduction of all involved lesion to normal size | FDG uptake Reduction DS= 4-5 | ≥ 50% reduction in SPD of up to 6 target lesions | Stable FDG uptake | No interval change | | Labelled IR7: - IR(1): >50% increase in SPD in first 12 weeks -IR(2): >50% increase in SPD with emergence of new lesion(s), or >50% increase in PPD of a lesion or set of lesions at any time during treatment -IR(3): Increase in FDG uptake without a concomitant increase in lesion size meeting criteria for PD | | Diagnosed retrospectively after achieving metabolic response following initial DP. | Accounting for pseudoprogression | Adding IR category to account for pseudoprogression |
| RECIL | FDG uptake normalization DS= 1-3. | Complete resolution of all target lesions and all nodes with LD8 < 1 cm | FDG uptake Reduction DS= 4-5 | ≥ 30% decrease in SLD9 of target lesions. | Any DS | Decrease < 10% to increase ≤ 20% in target lesions | | Any DS | > 20% increase in SLD | Same as LYRIC | Accounting for pseudoprogression | - Adding new category under the name of (Minor Response) associated with reduction in SLD between ≥ 10% and < 30%. -Progressive and minor response do not require correlation with Deauville criteria - Using Uni-dimensional, longest diameter of any target lesion to measure tumor burden |

## Slide 2
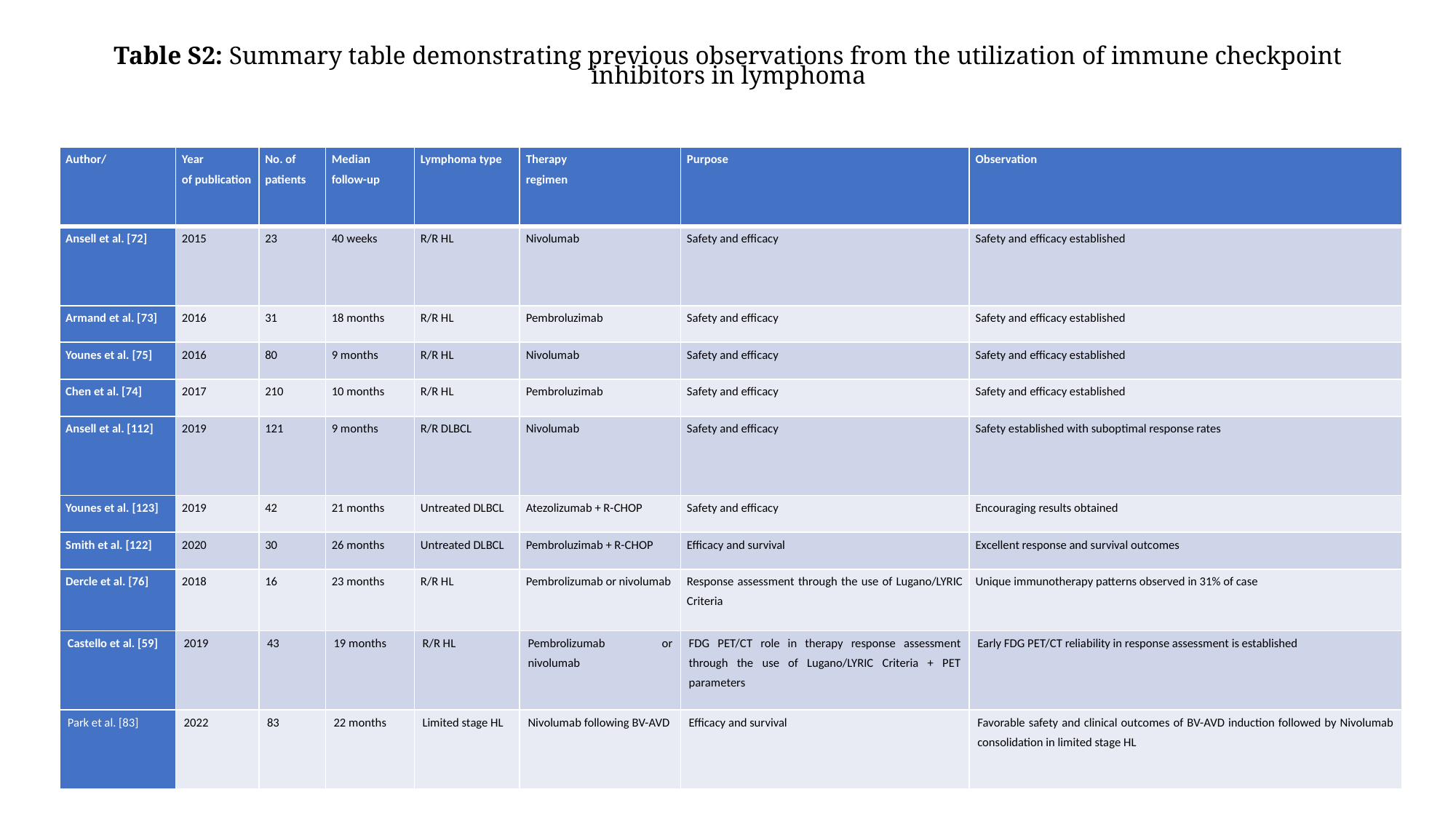

Table S2: Summary table demonstrating previous observations from the utilization of immune checkpoint inhibitors in lymphoma
| Author/ | Year of publication | No. of patients | Median follow-up | Lymphoma type | Therapy regimen | Purpose | Observation |
| --- | --- | --- | --- | --- | --- | --- | --- |
| Ansell et al. [72] | 2015 | 23 | 40 weeks | R/R HL | Nivolumab | Safety and efficacy | Safety and efficacy established |
| Armand et al. [73] | 2016 | 31 | 18 months | R/R HL | Pembroluzimab | Safety and efficacy | Safety and efficacy established |
| Younes et al. [75] | 2016 | 80 | 9 months | R/R HL | Nivolumab | Safety and efficacy | Safety and efficacy established |
| Chen et al. [74] | 2017 | 210 | 10 months | R/R HL | Pembroluzimab | Safety and efficacy | Safety and efficacy established |
| Ansell et al. [112] | 2019 | 121 | 9 months | R/R DLBCL | Nivolumab | Safety and efficacy | Safety established with suboptimal response rates |
| Younes et al. [123] | 2019 | 42 | 21 months | Untreated DLBCL | Atezolizumab + R-CHOP | Safety and efficacy | Encouraging results obtained |
| Smith et al. [122] | 2020 | 30 | 26 months | Untreated DLBCL | Pembroluzimab + R-CHOP | Efficacy and survival | Excellent response and survival outcomes |
| Dercle et al. [76] | 2018 | 16 | 23 months | R/R HL | Pembrolizumab or nivolumab | Response assessment through the use of Lugano/LYRIC Criteria | Unique immunotherapy patterns observed in 31% of case |
| Castello et al. [59] | 2019 | 43 | 19 months | R/R HL | Pembrolizumab or nivolumab | FDG PET/CT role in therapy response assessment through the use of Lugano/LYRIC Criteria + PET parameters | Early FDG PET/CT reliability in response assessment is established |
| Park et al. [83] | 2022 | 83 | 22 months | Limited stage HL | Nivolumab following BV-AVD | Efficacy and survival | Favorable safety and clinical outcomes of BV-AVD induction followed by Nivolumab consolidation in limited stage HL |

## Slide 3
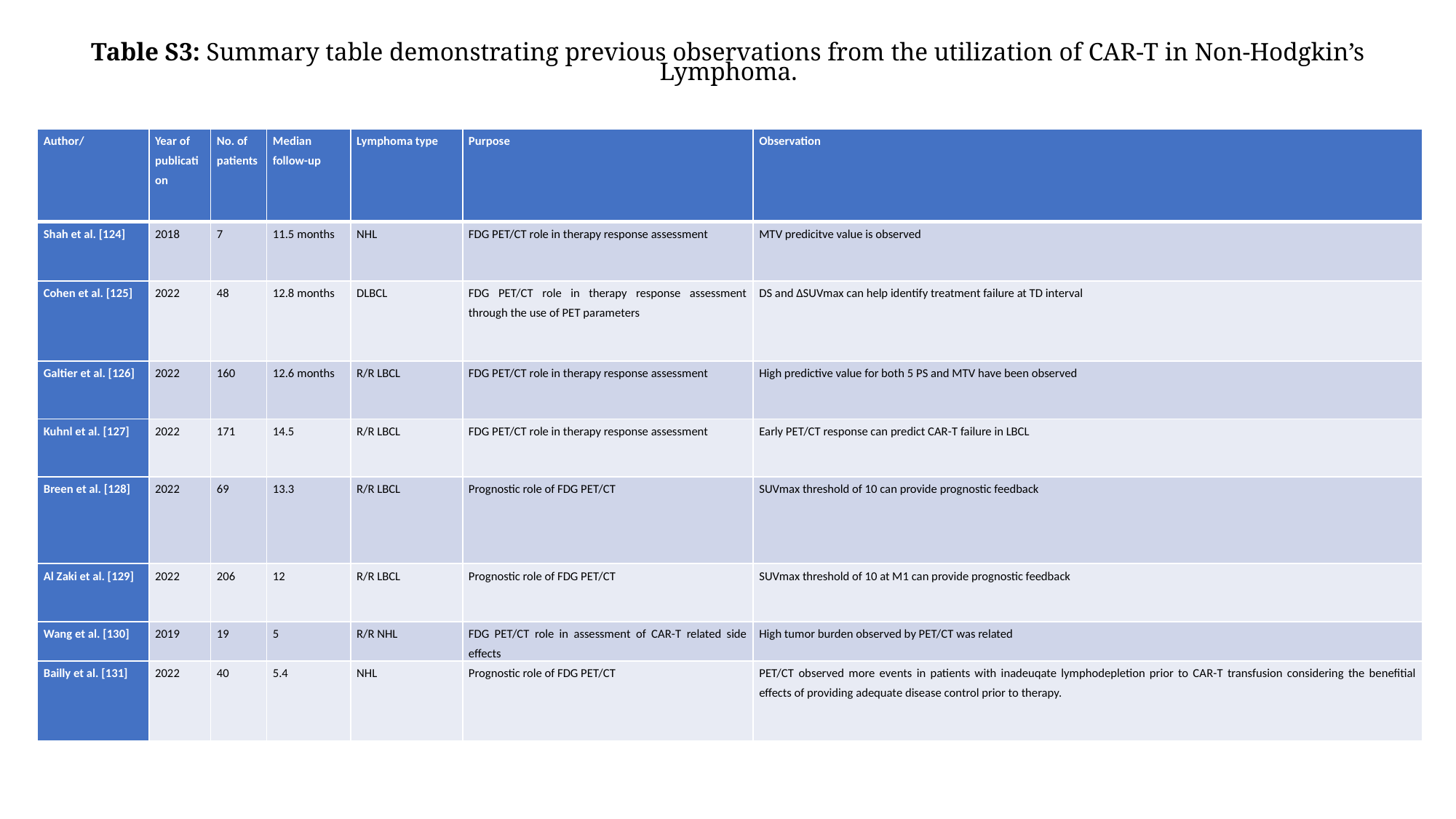

Table S3: Summary table demonstrating previous observations from the utilization of CAR-T in Non-Hodgkin’s Lymphoma.
| Author/ | Year of publication | No. of patients | Median follow-up | Lymphoma type | Purpose | Observation |
| --- | --- | --- | --- | --- | --- | --- |
| Shah et al. [124] | 2018 | 7 | 11.5 months | NHL | FDG PET/CT role in therapy response assessment | MTV predicitve value is observed |
| Cohen et al. [125] | 2022 | 48 | 12.8 months | DLBCL | FDG PET/CT role in therapy response assessment through the use of PET parameters | DS and ∆SUVmax can help identify treatment failure at TD interval |
| Galtier et al. [126] | 2022 | 160 | 12.6 months | R/R LBCL | FDG PET/CT role in therapy response assessment | High predictive value for both 5 PS and MTV have been observed |
| Kuhnl et al. [127] | 2022 | 171 | 14.5 | R/R LBCL | FDG PET/CT role in therapy response assessment | Early PET/CT response can predict CAR-T failure in LBCL |
| Breen et al. [128] | 2022 | 69 | 13.3 | R/R LBCL | Prognostic role of FDG PET/CT | SUVmax threshold of 10 can provide prognostic feedback |
| Al Zaki et al. [129] | 2022 | 206 | 12 | R/R LBCL | Prognostic role of FDG PET/CT | SUVmax threshold of 10 at M1 can provide prognostic feedback |
| Wang et al. [130] | 2019 | 19 | 5 | R/R NHL | FDG PET/CT role in assessment of CAR-T related side effects | High tumor burden observed by PET/CT was related |
| Bailly et al. [131] | 2022 | 40 | 5.4 | NHL | Prognostic role of FDG PET/CT | PET/CT observed more events in patients with inadeuqate lymphodepletion prior to CAR-T transfusion considering the benefitial effects of providing adequate disease control prior to therapy. |
