# Supplementary material for: FDG-PET/CT in the Monitoring of Lymphoma Immunotherapy Response: Current Status and Future Prospects
Source: Cancers (Basel). 2023 Feb 7;15(4):1063. doi: 10.3390/cancers15041063 (PMC9954669; doi:10.3390/cancers15041063)
Supplement: Supplementary file 1 [file cancers-15-01063-s001.zip › Updated Supplementary Material.pdf]

Table S1: Summary table demonstrating observed updates and modifications in therapy-specific Criteria applied since Lugano classification
[1]PET: Positron emission tomography; [2]CT: Computed tomography; [3]FDG: Fluorodeoxyglucose; [4]DS: Deauville score; [5]SPD: Sum of perpendicular diameters; [6]N/A: Not Applicable; [7]IR: Indeterminate response; [8]LD: Long diameter; [9]SLD: Sum of longest diameters.

Criteria	Complete Response		Partial Response		Stable Disease		Progression		Pseudoprogression	Value	Modifications
	PET[1]	CT[2]	PET	CT	PET	CT	PET	CT			
**Lugano**	FDG[3] uptake normalization DS= 1-3.	Reduction of all involved lesion to normal size	FDG uptake Reduction DS[4] = 4-5	≥ 50% reduction in SPD[5] of up to 6 target lesions	Stable FDG uptake	No interval change	- Metabolic progression with DS= 4-5<br>- New FDG-avid lesions	- ≥ 50% increase in SPD of lesions<br>- New lesion(s)	Not account for	Widely used	N/A[6]
**LYRIC**	FDG uptake normalization DS= 1-3.	Reduction of all involved lesion to normal size	FDG uptake Reduction DS= 4-5	≥ 50% reduction in SPD of up to 6 target lesions	Stable FDG uptake	No interval change	Labelled IR[7]:<br>- IR(1): >50% increase in SPD in first 12 weeks<br>-IR(2): >50% increase in SPD with emergence of new lesion(s), or >50% increase in PPD of a lesion or set of lesions at any time during treatment<br>-IR(3): Increase in FDG uptake without a concomitant increase in lesion size meeting criteria for PD		Diagnosed retrospectively after achieving metabolic response following initial DP.	Accounting for pseudoprogression	Adding IR category to account for pseudoprogression
**RECIL**	FDG uptake normalization DS= 1-3.	Complete resolution of all target lesions and all nodes with LD[8] < 1 cm	FDG uptake Reduction DS= 4-5	≥ 30% decrease in SLD[9] of target lesions.	Any DS	Decrease < 10% to increase ≤ 20% in target lesions	Any DS	> 20% increase in SLD	Same as LYRIC	Accounting for pseudoprogression	- Adding new category under the name of (Minor Response) associated with reduction in SLD between ≥ 10% and < 30%.<br>-Progressive and minor response do not require correlation with Deauville criteria<br>- Using Uni-dimensional, longest diameter of any target lesion to measure tumor burden

Table S2: Summary table demonstrating previous observations from the utilization of immune checkpoint inhibitors in lymphoma

Author/	Year of publication	No. of patients	Median follow-up	Lymphoma type	Therapy regimen	Purpose	Observation
Ansell et al. [72]	2015	23	40 weeks	R/R HL	Nivolumab	Safety and efficacy	Safety and efficacy established
Armand et al. [73]	2016	31	18 months	R/R HL	Pembroluzimab	Safety and efficacy	Safety and efficacy established
Younes et al. [75]	2016	80	9 months	R/R HL	Nivolumab	Safety and efficacy	Safety and efficacy established
Chen et al. [74]	2017	210	10 months	R/R HL	Pembroluzimab	Safety and efficacy	Safety and efficacy established
Ansell et al. [112]	2019	121	9 months	R/R DLBCL	Nivolumab	Safety and efficacy	Safety established with suboptimal response rates
Younes et al. [123]	2019	42	21 months	Untreated DLBCL	Atezolizumab + R-CHOP	Safety and efficacy	Encouraging results obtained
Smith et al. [122]	2020	30	26 months	Untreated DLBCL	Pembroluzimab + R-CHOP	Efficacy and survival	Excellent response and survival outcomes
Dercle et al. [76]	2018	16	23 months	R/R HL	Pembrolizumab or nivolumab	Response assessment through the use of Lugano/LYRIC Criteria	Unique immunotherapy patterns observed in 31% of case
Castello et al. [59]	2019	43	19 months	R/R HL	Pembrolizumab or nivolumab	FDG PET/CT role in therapy response assessment through the use of Lugano/LYRIC Criteria + PET parameters	Early FDG PET/CT reliability in response assessment is established
Park et al. [83]	2022	83	22 months	Limited stage HL	Nivolumab following BV-AVD	Efficacy and survival	Favorable safety and clinical outcomes of BV-AVD induction followed by Nivolumab consolidation in limited stage HL

Table S3: Summary table demonstrating previous observations from the utilization of CAR-T in Non-Hodgkin's Lymphoma.

Author/	Year of publication	No. of patients	Median follow-up	Lymphoma type	Purpose	Observation
**Shah et al. [124]**	2018	7	11.5 months	NHL	FDG PET/CT role in therapy response assessment	MTV predicitve value is observed
**Cohen et al. [125]**	2022	48	12.8 months	DLBCL	FDG PET/CT role in therapy response assessment through the use of PET parameters	DS and ΔSUVmax can help identify treatment failure at TD interval
**Galtier et al. [126]**	2022	160	12.6 months	R/R LBCL	FDG PET/CT role in therapy response assessment	High predictive value for both 5 PS and MTV have been observed
**Kuhnl et al. [127]**	2022	171	14.5	R/R LBCL	FDG PET/CT role in therapy response assessment	Early PET/CT response can predict CAR-T failure in LBCL
**Breen et al. [128]**	2022	69	13.3	R/R LBCL	Prognostic role of FDG PET/CT	SUVmax threshold of 10 can provide prognostic feedback
**Al Zaki et al. [129]**	2022	206	12	R/R LBCL	Prognostic role of FDG PET/CT	SUVmax threshold of 10 at M1 can provide prognostic feedback
**Wang et al. [130]**	2019	19	5	R/R NHL	FDG PET/CT role in assessment of CAR-T related side effects	High tumor burden observed by PET/CT was related
**Bailly et al. [131]**	2022	40	5.4	NHL	Prognostic role of FDG PET/CT	PET/CT observed more events in patients with inadeuqate lymphodepletion prior to CAR-T transfusion considering the benefitial effects of providing adequate disease control prior to therapy.
